# Ionic Liquid–Glycol Mixtures for Direct Air Capture of CO_2_: Decreased Viscosity and Mitigation of Evaporation Via Encapsulation

**DOI:** 10.1021/acssuschemeng.4c01265

**Published:** 2024-05-07

**Authors:** Cameron D. L. Taylor, Aidan Klemm, Luma Al-Mahbobi, B. Jack Bradford, Burcu Gurkan, Emily B. Pentzer

**Affiliations:** † Department of Materials Science and Engineering, Texas A&M University, College Station, Texas 77843, United States; ‡ Department of Chemical Engineering Biomolecular Engineering, Case Western Reserve University, Cleveland, Ohio 44106, United States; § Department of Chemistry, Texas A&M University, College Station, Texas 77843, United States

**Keywords:** direct air capture, CO_2_ sorbent encapsulation, viscosity reduction, ionic liquid, volatility reduction

## Abstract

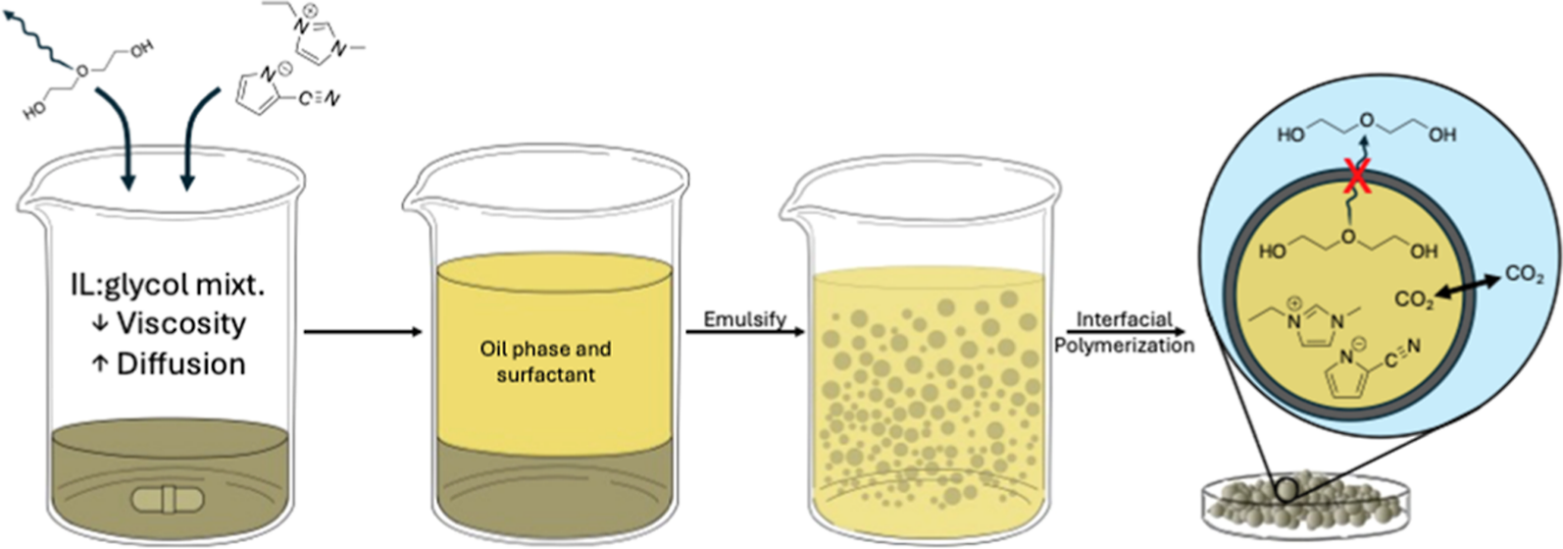

Herein we address the efficiency of the CO_2_ sorption of ionic liquids (IL) with hydrogen bond donors (e.g., glycols) added as viscosity modifiers and the impact of encapsulating them to limit sorbent evaporation under conditions for the direct air capture of CO_2_. Ethylene glycol, propylene glycol, 1,3-propanediol, and diethylene glycol were added to three different ILs: 1-ethyl-3-methylimidazolium 2-cyanopyrrolide ([EMIM][2-CNpyr]), 1-ethyl-3-methylimidazolium tetrafluoroborate ([EMIM][BF_4_]), and 1-butyl-3-methylimidazolium tetrafluoroborate ([BMIM][BF_4_]). Incorporation of the glycols decreased viscosity by an average of 51% compared to bulk IL. After encapsulation of the liquid mixtures using a soft template approach, thermogravimetric analysis revealed average reductions in volatility of 36 and 40% compared to the unencapsulated liquid mixtures, based on 1 h isothermal experiments at 25 and 55 °C, respectively. The encapsulated mixtures of [EMIM][2-CNpyr]/1,3-propanediol and [EMIM][2-CNpyr]/diethylene glycol exhibited the lowest volatility (0.0019 and 0.0002 mmol/h at 25 °C, respectively) and were further evaluated as CO_2_ absorption/desorption materials. Based on the capacity determined from breakthrough measurements, [EMIM][2-CNpyr]/1,3-propanediol had a lower transport limited absorption rate for CO_2_ sorption compared to [EMIM][2-CNpyr]/diethylene glycol with 0.08 and 0.03 mol CO_2_/kg sorbent, respectively; however, [EMIM][2-CNpyr]/diethylene glycol capsules exhibited higher absorptions capacity at ∼500 ppm of CO_2_ (0.66 compared to 0.47 mol of CO_2_/kg sorbent for [EMIM][2-CNpyr]/1,3-propanediol). These results show that glycols can be used to not only reduce IL viscosity while increasing physisorption sites for CO_2_ sorption, but also that encapsulation can be utilized to mitigate evaporation of volatile viscosity modifiers.

## Abstract 2

This study evaluates the use of soft-template encapsulation to suppress glycol evaporation in ionic liquid–glycol mixtures, accelerating direct air capture of CO_2_ in ionic liquids by both viscosity reduction and gas−liquid surface area augmentation.

## Introduction

As atmospheric CO_2_ concentrations continue to rise, alternative approaches for negative emissions technologies (NET), such as carbon capture, must be implemented in complement to decarbonization efforts to avoid the average global surface temperature from rising greater than 2 °C by the mid-21st century.^1^ One NET approach is direct air capture (DAC), which utilizes chemical reactions and physical interactions to sequester CO_2_ from the atmosphere. DAC is more energy intensive compared to point source CO_2_ capture due to the lower concentration of CO_2_ in the atmosphere (∼420 ppm for DAC vs > 10,000 ppm for point source).^2^ To date, DAC approaches have focused on liquid solvents^3^ (e.g., aqueous amines), solid sorbents^4^ (e.g., metal organic frameworks, MOFs), and pressure driven approaches^5^ (e.g., membranes). Current technologies in industrial DAC applications are hindered by low CO_2_ sorption rates and capacity, high energy demands for sorbent regeneration, and poor scalability.^6^

Solvent-based chemical DAC technologies typically require strong bases due to the low concentration of CO_2_ in the atmosphere. For example, aqueous solutions of NaOH and KOH are common in DAC applications, in addition to amines and amine-functionalized molecules (e.g., amino acids).^3,6^ Strong bases typically chemisorb CO_2_, for example, with a hydroxide anion reacting to form bicarbonate and carbonate anions. Although this can be favorable for the capture of CO_2_, desorption is highly endothermic; for example, temperatures of ∼900 °C are required for calcination of CaCO_3_ to CaO. Aqueous amines, such as monoethanolamine, are a significantly developed liquid sorbent class for point-source CO_2_ capture (i.e., flue gas) and have been applied to DAC, but they pose significant challenges because the volatile amines evaporate into the effluent air stream and more so into the captured CO_2_ stream, leading to corrosion and requiring additional energy for recondensation and scrubbing. Less volatile alternatives to aqueous bases and amines include solvents like aqueous amino acids and ionic liquids (ILs) which typically have decreased regeneration temperatures of 70–120 °C.^3,7^

ILs have negligible volatility, chemical and thermal stability, and tunability making them attractive candidates for DAC.^7^ Conventional ILs, such as 1-butyl-3-methylimidazolium hexafluorophosphate [BMIM][PF_6_], primarily rely on physisorption of CO_2_ into free molar volume.^8,9^ By tailoring the functional moieties, task specific ILs (TSILs) can absorb CO_2_ via chemisorption, as well as physisorption,^10^ offering a promise as DAC solvents. For example, the Brennecke group developed the TSIL 1-ethyl-3-methylimidazolium 2-cyanopyrrolide ([EMIM][2-CNpyr]),^11^ and the Gurkan group demonstrated DAC abilities.^12,13^ The Gurkan group determined two reaction routes are possible for [EMIM][2-CNpyr] DAC: the anion reversibly reacts with CO_2_ to form carbamate and the cation reacts to form imidazolium-carboxylate.^14^ Notably, many TSILs are currently not economically viable for broad implementation in DAC operations compared to current solvents (e.g., aqueous amines, KOH, etc.) due to synthetic costs.^15,16^

**Figure 1:**
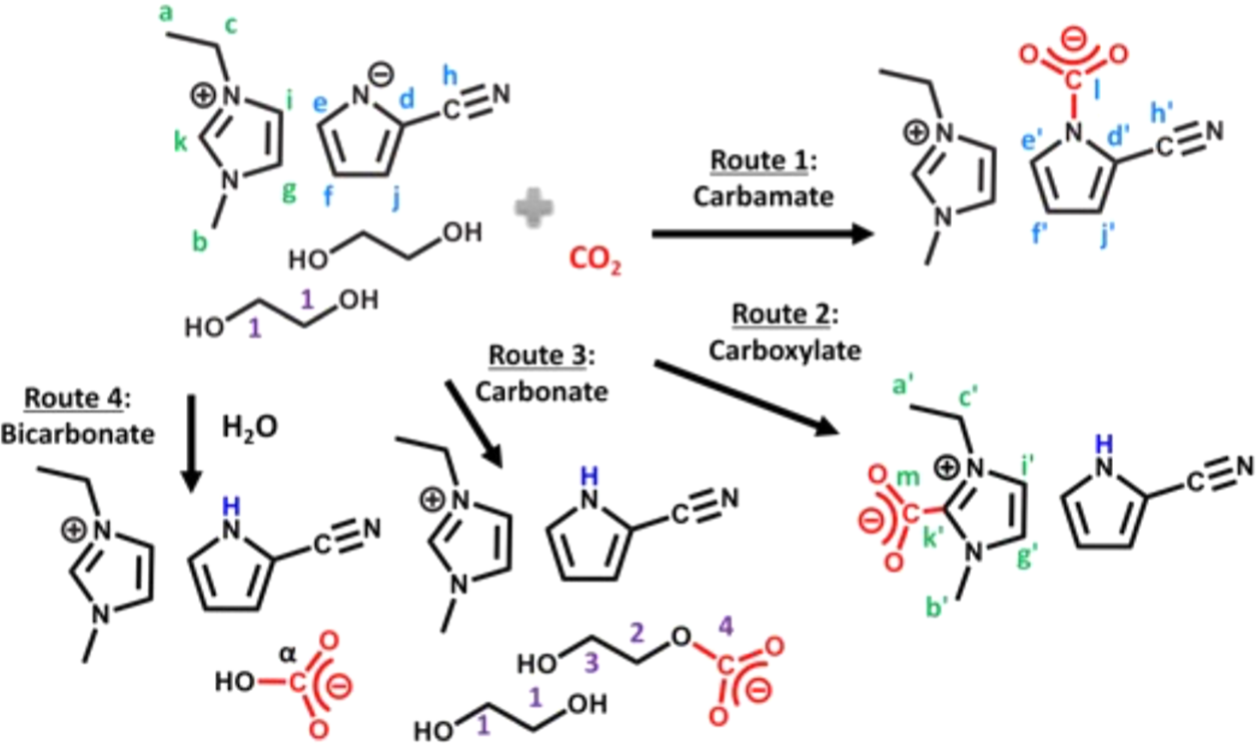
Proposed reaction pathways of CO_2_ with [EMIM][2-CNpyr]/EG (1:2). Reproduced from ref (19) with permission from the American Chemical Society (2021).

Applications of TSILs are commonly limited by high viscosity, primarily during the absorption of CO_2_,^17^ which can slow the rate of CO_2_ absorption,^15^ thereby making the processing of large volumes of air for CO_2_ removal a challenge. TSILs for DAC are further limited due to solvent pumping costs, which increase as viscosity increases. One approach to improve CO_2_ absorption rates is mixing TSILs with additives to reduce viscosity.^18,19^ These solvent systems can also reduce the quantity of IL required and supply additional CO_2_ binding motifs to enhance the sorption rate and capacity. For instance, Camper et al. showed that mixing imidazolium-based ILs (e.g., [HMIM][Tf_2_N]) with amines (e.g., monoethanolamine) improved CO_2_ absorption by at least 20 times compared to neat IL due to an increase in chemisorption capacity.^20^ Meanwhile, Nookuea et al. demonstrated that adding a hydrogen bond donor (HBD) (e.g., monoethanolamine) can reduce the overall viscosity of the mixture.^21^ The molecular interactions between ILs and other additives have been studied, suggesting that strong intermolecular interactions (e.g., hydrogen bonding) reduce the evaporation of the additive as well as improve CO_2_ sorption.^19,22^ For example, Lee et al. demonstrated that a 1:2 IL/HBD molar mixture of [EMIM][2-CNpyr] and ethylene glycol (EG) had improved absorption rate of CO_2_ which was attributed to reduced viscosity compared to the pure IL.^19^ The authors also determined that EG aided in CO_2_ sorption by protonating the anion and forming a complex with CO_2_, as seen in Route 3 of Figure 1. Importantly, increasing the loading of EG (i.e., IL/EG at 1:3) resulted in noticeable evaporative loss of EG, while lower compositions resulted in thermal stability, attributed to complexation of EG with CO_2_.

Implementation of ILs for DAC is not limited to traditional solvent-based approaches (i.e., bulk liquids), and in complement can be used in various composite structures such as membranes (e.g., polymer-IL, supported IL membranes, and cross-linked gels),^23−25^ IL-impregnation in porous structures (e.g., silica),^26−29^ and microencapsulation.^14,30−35^ In many of these composites, the gas-contacting surface area of the IL is greatly increased compared to that of bulk systems, which can minimize the diffusion limitations posed by the high IL viscosity. Of these composite structures, encapsulation is perhaps the most scalable and most flexible for different compositions. Three main approaches can be used to encapsulate ILs: microfluidics,^36^ hard template approach,^37^ or soft template approach, of which the soft template approach is the most common. Here, a shell is grown around a droplet of the desired core, typically relying on an emulsion, with liquid droplets stabilized by surfactants. The encapsulation of different types of ILs have been studied for postcombustion capture of CO_2_ and for DAC. For example, Bernard et al. encapsulated imidazolium based fluorinated ILs from an emulsion.^38^ The Pentzer Group encapsulated a variety of ILs utilizing the soft template approach and emulsion stabilized with modified graphene oxide;^39−41^ the resulting capsules of IL had applications in toxin removal,^42−44^ CO_2_ capture,^14,45,46^ protecting phase change materials,^47^ and payload release.^48−50^ In complement, the Gurkan group developed different deep eutectic solvents, such as those based on choline for CO_2_ sorption,^51^ and studied the kinetics and intermolecular interactions of the TSILs for CO_2_ sorption.^13,52^ In collaboration between the two groups, the versatility of the encapsulation techniques enables the applicability of more volatile additives for CO_2_ sorbents by having the shell act as a container for the solvent while being CO_2_ permeable. Currently, limited studies address the role of different glycols in CO_2_ sorption and modifying viscosity of ILs,^53−55^ and little is understood how encapsulation can reduce evaporation of volatile compounds (e.g., glycols).^56^

Herein, we demonstrate that glycol-based HBD additives beyond EG can be used to decrease the viscosity of ILs by up to 50% and that the volatility of the glycols can be reduced by 36 and 40% compared to that of pure glycols, respectively, at DAC-relevant temperatures (e.g., 25 and 55 °C). The HBDs used are EG, propylene glycol (PG), 1,3-propanediol (1,3-P), and diethylene glycol (DEG) and were mixed with ILs ([EMIM][BF_4_], and [BMIM][BF_4_]) and TSIL ([EMIM][2-CNpyr]). Further reductions in volatility were achieved by encapsulating these liquid mixtures with reductions of 40 and 43% at 25 and 55 °C, respectively, compared to pure glycols. We further demonstrate that encapsulation enhances CO_2_ sorption rates of the IL/glycol mixtures, with [EMIM][2-CNpyr]/1,3-P having the fastest uptake rate compared to [EMIM][2-CNpyr]/DEG (0.65 and 0.34 mmol CO_2_, respectively). The use of DEG and 1,3-P as viscosity modifiers increased the CO_2_ capacity by factors of 3 and 4, respectively. The least volatile samples ([EMIM][2-CNpyr]/1,3-P and [EMIM][2-CNpyr]/DEG) were studied for CO_2_ sorption applications, demonstrating promising performance compared with the neat ILs themselves. With the use of viscosity modifiers and encapsulation, TSILs can be more widely utilized in a variety of applications such as DAC as well as e.g., wastewater treatment, pharmaceuticals, and consumer goods.

## Methods

### Materials

The IL, 1-ethyl-3-methylimidazolium 2-cyanopyrrolide ([EMIM][2-CNpyr]), with a purity of >95%, was synthesized as previously reported.^14^ 1-Ethyl-3-methylimidazolium tetrafluoroborate ([EMIM][BF_4_]) (>98%) and 1-butyl-3-methylimidazolium tetrafluoroborate ([BMIM][BF_4_]) (>98%) were purchased from Iolitec and were dried at 80 °C under reduced pressure for 72 h; [EMIM][2-CNpyr] was dried under reduced pressure at 70 °C for 72 h to avoid thermal decomposition. All other materials were used as received. Graphite flakes, propylamine, potassium permanganate, 4,4′-diaminodiphenylmethane (DAPM), hexamethylene diisocyanate (HDI), diethylene glycol (DEG), 1,3-P, and sulfuric acid (95–98%) were purchased from Sigma-Aldrich. Hexanes, hydrogen peroxide (35 wt % in water), and toluene were purchased from Fisher Scientific. Additionally, *n*-octane (Oakwood Chemical), EG (Acros Organics), PG (TCI), and *N*,*N*-dimethylformamide (DMF, Alfa Aesar) were all purchased and used as received. Nitrogen gas (Ultra High Purity) and carbon dioxide gas (Bone Dry) were purchased from Airgas.

### Instrumentation

Mixture composition and capsule core loading were characterized by nuclear magnetic resonance (NMR) spectroscopy in DSMO-*d*_6_ using a Bruker Avance NEO 400 mHz NMR spectrometer. Centrifugation was conducted using a ThermoScientific Sorvall ST 8 centrifuge. Bath ultrasonication was performed with a Fisherbrand CPX3800 5.7 L Ultrasonic Bath. Emulsification was done using a BioSpec hand-held homogenizer, model 985370. The capsule morphology was analyzed using scanning electron microscopy (Tescan Vega SEM) at a voltage of 10–20 kV; prior to characterization, the sample was sputter-coated with 10 nm of Au (Cressington 108 Sputter Coater). Fourier transform infrared (FTIR) spectroscopy was performed on a JASCO FT/IR-4600 using a diamond coated ZnSe crystal in the ATR mode. Thermogravimetric analysis (TGA) was performed on a TA Instruments TGA 5500 equipped with a TA Instruments Blending Gas Delivery Module under N_2_ and bone-dry CO_2_. Viscosity measurements were performed using an Anton Paar MCR-302 rotational rheometer with an Anton Paar 25 mm 0.5° cone CP25-0.5 top plate and an Anton Paar 24.986 mm parallel plate. The density of the mixtures was collected by using an Anton Paar vibrating U-tube density meter (DMA 4500M) with an accuracy of 0.00005 g/cm^3^.

### IL/Glycol Mixture Preparation and Characterization

Mixtures of IL and glycols were prepared with molar ratios of 1:2, and confirmed by ^1^H NMR spectroscopy using DMSO-*d*_6_ as the solvent.

### Synthesis of Graphene Oxide

Graphene oxide (GO) nanosheets were synthesized using a modified Hummer’s method, as previously reported.^57^ Briefly, graphite flakes (3 g) were dispersed in concentrated H_2_SO_4_ (400 mL) at room temperature. KMnO_4_ (3 g) was slowly added, and after complete addition the mixture was stirred at room temperature for 24 h; this was repeated 3 times for a total addition of 12 g of KMnO_4_. Then, after stirring for a total of 72 h, the reaction was quenched by adding the solution into three Erlenmeyer flasks each containing ∼750 mL of ice water. Dropwise addition of H_2_O_2_ to the stirred solution was continued until the color turned from pink to brown, indicating excess KMnO_4_ was consumed. The yellow-brown GO was isolated by centrifugation and subsequent washing of the pellet with isopropyl alcohol, each time discarding the supernatant and repeating until the supernatant was neutral by a litmus test. The GO was then dried overnight under reduced pressure at room temperature and thereafter blended to a fine powder. The powder was stored in a refrigerator and sealed with parafilm.

### Synthesis of Alkylated GO

Alkylated GO (C_18_-GO) was synthesized following a method previously reported.^39^ GO (100 mg) was dispersed in DMF (40 mL) via sonication, until no visible aggregates were observed. Meanwhile, octadecylamine (900 mg) was dissolved in DMF (60 mL) by gently heating to 60 °C in a 250 mL round-bottomed flask (rbf). The GO solution was added to the rbf and the resulting mixture stirred at 55 °C for ∼5 min. The sample was then centrifuged, and the supernatant was discarded. The pellet was suspended in toluene (40 mL) via sonication. In a separate rbf, octadecylamine (2.7 g) was dissolved in toluene (60 mL) while being stirred at 60 °C. Once the octadecylamine was dissolved, the GO dispersion was added, and the mixture was stirred at 55 °C overnight. Thereafter, a dark brown precipitate was isolated via centrifugation, washed with octane (2 × 25 mL, discarding the supernatant each time), and dried under reduced pressure at ambient temperature. When ready to use, ∼100 mg of C_18_-GO was dispersed in 1:1 v/v mixture of octane (25 mL) and heavy mineral oil (25 mL), resulting in a C_18_-GO concentration of 2 mg/mL.

### Synthesis and Characterization of Microcapsules

Based on a previously reported method,^40^ capsules were synthesized by interfacial polymerization in an emulsion (i.e., a soft template approach). As an example, take the preparation of capsules with a core of pure EG. First, DAPM (0.66 mmol, 130.8 mg) was dissolved in 0.5 mL of EG in a 20 mL scintillation vial via sonication. Then, C_18_-GO in octane/mineral oil (2.5 mL of a 2 mg/mL solution) was added to the EG/DAPM solution and emulsified by 3 cycles of shear mixing (20 s on, 15 s off), which produced an emulsion with droplets of EG/DAPM in a continuous phase of octane and mineral oil. The prepared emulsion was diluted with octane (1 mL). In a separate vial, HDI (0.86 mmol, 137.8 μL) was mixed with octane (1.25 mL) and this solution was added dropwise to the emulsion by swirling the vial by hand. The system was left unagitated at ambient temperature for 72 h, and then the capsules were isolated via gravity filtration and washed with hexanes (∼100 mL) and then dispersed in hexanes (100 mL). Residual isocyanate groups were quenched by adding propylamine (2 mL) to the dispersion and allowing the system to rest for 1–2 h. Finally, the capsules were isolated via gravity filtration and washed with hexanes until pH of effluent is about neutral to verify propylamine removal (∼300 mL of hexanes). The capsules were air-dried for an hour. A similar procedure was used for all capsules, with the discontinuous phases being ILs, glycols, or IL/glycol mixtures. The amount of monomer was consistent across all the samples, excluding the [EMIM][2-CNpyr] capsules, for which a second additional HDI was used (0.43 mmol), with a total loading of 1.29 mmol HDI.

The loading of the core liquid in the capsules was determined by extraction of the core using DSMO-*d*_6_ and mesitylene standard, and characterization by ^1^H NMR, as previously reported.^39^ Capsules (∼20 mg) were weighed in a glass vial, and then a 0.038 M mesitylene in DMSO-*d*_6_ solution (1 mL) was added. The sample was sonicated for ∼3 min to extract the core liquid and then passed through a PTFE syringe filter to separate the solid capsule shell. Relative integration of the ^1^H NMR signals due to mesitylene and the IL, glycol, or IL and glycol was used to determine the wt % of the core in the capsule (Figure S1). Capsule sizing was done using ImageJ and SEM images with 100–500 capsules per sample.

### Evaporation Rate Measurements

Evaporation rate studies were determined using thermogravimetric analysis (TGA) under an inert N_2_ environment. As an example, bulk EG (20 μL) was spread on a tared, flame-cleaned high-temperature platinum TGA pan. A 1 h isothermal measurement under N_2_ (flow rate of 25 mL/min) was conducted at 25 °C, monitoring the mass. The sample pan was cleaned, a fresh sample of EG (20 μL) was added, and a 1 h isothermal measurement under N_2_ (flow rate of 25 mL/min) at 55 °C was collected. A similar procedure was used on all unencapsulated pure glycols, pure ILs, and IL/glycol mixtures. Samples containing 1,3-P and DEG were pretreated at 55 °C under N_2_ (flow rate of 25 mL/min) for 5 min to desorb any volatiles resulting in a stable mass loss at 25 °C.

The evaporation rates of the encapsulated liquids were determined by using the method described above. To compare capsules to bulk samples, the molar quantities of the liquids were held constant by using the core wt % loading of the capsules. For example, 20 μL of bulk EG has 0.358 mmol of EG and the capsules of EG had a measured core loading of 60 ± 3 wt % EG. Therefore, the amount of EG capsules used was 37 mg, using
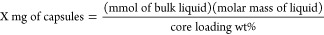
1

The thermal stability of the encapsulated and unencapsulated samples were determined using TGA with heating ramped from ambient temperature to 500 °C by 10 °C/min under N_2_ (25 mL/min) (Figure S2).

### CO_2_ Sorption Studies

The IL/glycol mixtures with the lowest evaporation rates (i.e., [EMIM][2-CNpyr]/1,3-P and [EMIM][2-CNpyr]/DEG) were evaluated for CO_2_ sorption by utilizing both thermogravimetric and breakthrough techniques.

Thermogravimetric CO_2_ sorption studies were conducted using TGA with flow rates of both CO_2_ and N_2_ at 25 mL/min. On a clean platinum TGA pan, ∼10 mg of capsules was loaded. The capsules were pretreated to remove any absorbed gases and moisture at 55 °C under N_2_ (25 mL/min) until the mass of the sample exhibited a similar linear mass loss determined from the evaporation studies, as previously determined. Then, the sample was cooled from 55 to 25 °C at a rate of 10 °C/min and a baseline mass was established. Then, N_2_ was switched to CO_2_ (25 mL/min). The mass of the sample increased steadily as the CO_2_ was absorbed until a plateau was reached; then, for desorption, the process gas was switched back to N_2_ (25 mL/min) and the temperature was increased to 55 °C (10 °C/min). As the CO_2_ was desorbed, a decrease in mass was observed and the mass loss was stabilized at approximately the baseline as established with the pretreatment. This CO_2_ sorption–desorption cycle was completed 10 times for both capsule samples.

Breakthrough experiments were performed following a previously described procedure.^14^ Briefly, capsules (0.25 ± 0.01 g) were lightly packed into a 0.305 in. inner diameter column, with care taken not to break the capsules. The column was purged at 55 °C in a temperature-controlled incubator (HettCube 400R; Across International LLC) with pure N_2_ (Ultra High Purity, Airgas) at 50 standard cm^3^/min (sccm) utilizing a mass flow controller (Brooks i5850, 0–200 sccm) and the effluent gas was analyzed by an infrared gas analyzer (SBA-5, PP Systems, Inc.), abbreviated IRGA. Once CO_2_ signal reached 0 ppm, the incubator was cooled to 25 °C. A mixture of 502 ± 5 ppm of CO_2_ in N_2_ (custom gas mixture, Airgas) was fed to a bypass to calibrate the IRGA and the gas feed composition was confirmed to be stable. After 1 min, the feed gas was diverted to the sample column, and the effluent CO_2_ concentration was measured over time. The experiment was stopped when the concentration of CO_2_ in the effluent reached the feed concentration of 502 ± 5 ppm. Capacity analysis was performed by integrating the breakthrough curve and using the following equation
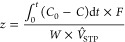
2where *z* is the CO_2_ loading (mmol CO_2_/g sorbent). *C*_0_ is the dimensionless feed CO_2_ composition (concentration in ppm × 10^6^), *C* is the dimensionless effluent CO_2_ composition, *t* is time (min), *F* is the total mass flow rate of the feed gas (sccm), *W* is the weight of the sample (g), and *V̂*_STP_ is the molar volume of CO_2_ at STP (22.4 sccm CO_2_/mmol CO_2_, assuming ideal gas law in dilute conditions). Breakthrough time (*t*_BT_) and pseudoequilibrium time (*t*_PE_) are defined as the time at which the effluent concentration reached 25 ppm of CO_2_ (5% of the feed concentration) and 490 ppm (97.5% of the feed concentration), respectively.

## Results and Discussion

### Materials Selection, Preparation, and Characterization

Four different glycols were used as viscosity modifiers for three different ILs, each chosen for their commercial availability or ease of accessibility. A total of 12 IL/glycol compositions were prepared, each with a 1:2 molar ratio of IL to glycol, based on prior studies.^19,58^ Two common ILs ([EMIM][BF_4_] and [BMIM][BF_4_]) and one TSIL ([EMIM][2-CNpyr]) were used. [EMIM][BF_4_] and [BMIM][BF_4_] absorb CO_2_ via physisorption into free volume and differ by the length of one alkyl chain on the imidazolium cation resulting in a difference in viscosities.^25^ In contrast, [EMIM][2-CNpyr] chemisorbs CO_2_ via covalent binding (Figure 1).^14,19,59,60^ The four glycols were chosen as low-cost and noncorrosive viscosity modifiers with the ability to hydrogen bond through their hydroxyl groups: EG, PG, 1,3-P, and DEG. Thus, all mixtures were homogeneous at room temperature, apart from [EMIM][BF_4_]/1,3-P and [BMIM][BF_4_]/1,3-P which phase separated at room temperature but formed a homogeneous solution at 29 and 26 °C, respectively.

**Figure 2:**
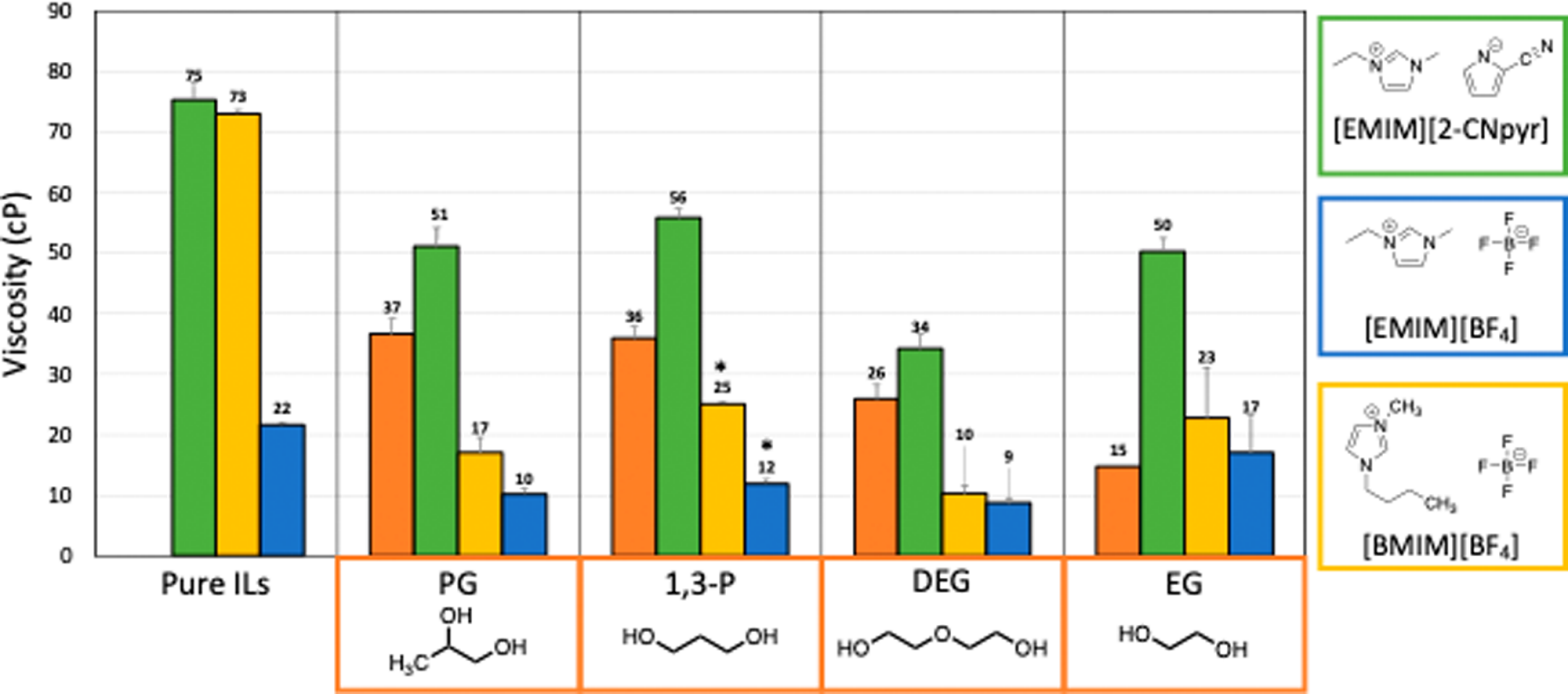
Viscosity of pure ILs and their mixtures for [EMIM][2-CNpyr] (green), [EMIM][BF_4_] (blue), [BMIM][BF_4_] (yellow), and pure glycols (orange). Data were collected at 25 °C except samples marked with an “*” which were taken at 40 °C.

Figure 2 shows the viscosity of the pure ILs, pure glycols, and their mixtures at 25 °C (with the exception for [EMIM][BF_4_]/1,3-P and [BMIM][BF_4_]/1,3-P which were recorded at 40 °C). The viscosities of the pure ILs were: 75.3 ± 2.1 cP for [EMIM][2-CNpyr], 21.6 ± 6.2 cP for [EMIM][BF_4_], and 73.0 ± 8.5 cP for [BMIM][BF_4_]. Despite having the same cation, [EMIM][2-CNpyr] and [EMIM][BF_4_] display significantly different viscosities due to the different polarities of the anions. Interestingly, the viscosities of [EMIM][2-CNpyr] and [BMIM][[BF_4_] were similar, yet [BMIM][BF_4_] had a more significant viscosity reduction with the addition of glycol modifiers. All IL/glycol mixtures had decreased viscosity compared to the pure ILs, with [EMIM][BF_4_] and [BMIM][BF_4_] mixtures also having a viscosity lower than that of the pure glycols, suggesting that the formation of the mixture interrupts intermolecular interactions (e.g., a eutectic forms). In contrast, [EMIM][2-CNpyr] mixtures were more viscous compared to the corresponding pure glycols, indicating strong interactions between the glycols and the IL. The water content of the mixtures was measured to be between 1900 and 7600 ppm, with EG mixtures having the highest water content (7300–7600 ppm) and the other mixtures having a lower water content: PG (2100–2200 ppm), 1,3-P (2900–3900 ppm), and DEG (1900–2700 ppm). All water content data can be found in Table S1.

To further examine the molecular interactions of the mixture, excess molar volume was calculated using eq 3
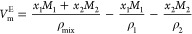
3

where ρ is density, *x* is mole fraction, and M is molar mass. Values are reported in Table S1. The negative excess molar volumes for all [EMIM][2-CNpyr]–glycol mixtures indicate compacting of the liquid, further supporting that hydrogen bond network formed between the IL and the glycol, thereby resulting in a closer packing of the molecules, thus ahigher viscosity.^61^ On the other hand, [EMIM][BF_4_]– and [BMIM][BF_4_]–glycol mixtures had positive excess molar volumes, indicating that the constituent species are not strongly interacting, resulting in lower viscosities. Although the analysis of the mixtures of [EMIM][BF_4_] and [BMIM][BF_4_] with 1,3-P used the densities at 40 °C, the excess molar volume is still very large and positive (3.9 and 2.1 mL/mol, respectively). These large positive values suggest weakened molecular interactions, which is reasonable due to the mixture phase separating at 25 °C. The excess molar volume results suggest the following IL trend in strength of intermolecular interactions with glycols: [EMIM][2-CNpyr] > [BMIM][BF_4_] > [EMIM][BF_4_].

**Figure 3:**
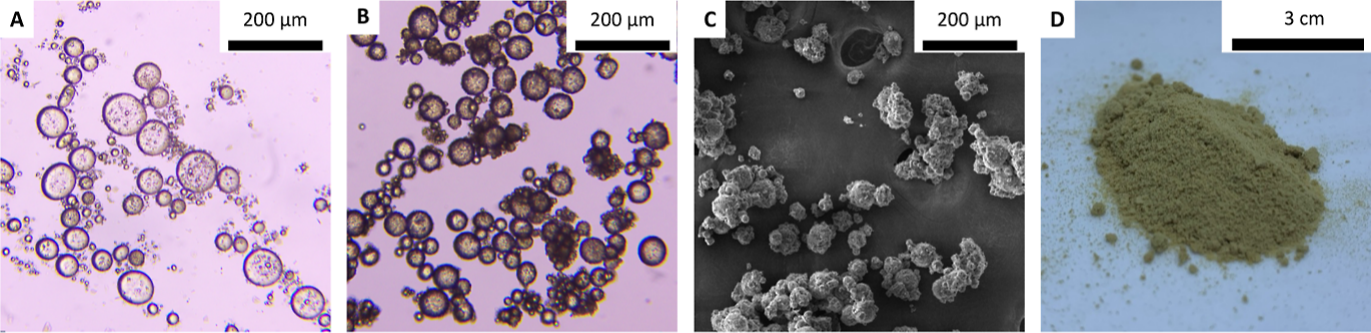
Images of the [EMIM][2-CNpyr]/1,3-P emulsion and capsules: (A) Optical microscopy image of emulsion showing discrete IL/glycol droplets in a mineral oil/octane continuous phase, (B) optical microscopy image of capsules before isolation with visible polyurea shell formation, (C) SEM image of isolated capsules, (D) photograph of the isolated capsules post air-drying for 1 h.

To increase the surface area for DAC, encapsulation of the pure ILs, pure glycols, and their mixtures was accomplished using a soft-template approach. Briefly, the liquid for the core was shear mixed with an octane/mineral oil dispersion of C_18_-GO nanosheets (see the Synthesis and Characterization of Microcapsules section for details) to form a nanosheet-stabilized Pickering emulsion (Figure 3A). By preloading the droplet with 4,4′-diaminodiphenylmethane (DAPM) and adding hexamethylene diisocyanate (HDI) into the octane phase of the emulsion, A2 + B2 step growth interfacial polymerization of the diamine and diisocyanate yielded a polyurea capsule shell around the droplet (Figure 3B,C). The capsules were isolated by gravity filtration then residual isocyanate groups were quenched with propyl amine. The capsules were air-dried for 1 h, yielding solid capsules in gram scale (Figure 3D); emulsion droplets were slightly smaller than the corresponding capsules, assumedly due to the polymer shell formation around the droplet (i.e., polymerization took place in the outer part of the droplet).

Images of all emulsions and capsules are shown in Figures S3 and S4, respectively, and illustrate spherical emulsion droplets and capsules tens of micrometers in diameter. The size distribution for each capsule system was evaluated by utilizing SEM images and ImageJ analysis of 100–500 capsules per sample (Table S1). Overall, the average diameter sizes of the capsules with [EMIM][BF_4_] mixtures had the smallest average diameter (23 ± 9 μm), [BMIM][BF_4_] mixtures had an average of 42 ± 20 μm, and capsules of [EMIM][2-CNpyr] mixtures had the largest average diameter (52 ± 15 μm). The largest capsules were pure DEG and [EMIM][2-CNpyr]/DEG, 70 ± 29 and 70 ± 16 μm, respectively.

The core loading wt % of the capsules was determined by extracting the core liquid using a deuterated solvent containing an internal standard and characterization of the liquid by ^1^H NMR spectroscopy (a representative spectrum is shown in Figure S1). The spectra were analyzed by integrating signals from each constituent molecule present and comparing them to the internal standard. The average core wt % across all samples was 52 ± 2 wt %. For example, capsules of [EMIM][BF_4_] and its mixtures had an average loading of at 62 ± 1 wt % whereas capsules of [BMIM][BF_4_] and its mixtures had an average loading of 60 ± 1 wt %. However, capsules of [EMIM][2-CNpyr] and its mixtures had the lowest average loading of 31 ± 2 wt %, which may be due to the thicker shell (i.e., to prepare isolable capsules additional monomer was used). A negative correlation was observed between the core viscosity and loading. Capsules with higher viscosity cores resulted in a lower wt % of core material; for example, [EMIM][2-CNpyr] (viscosity of 75.3 ± 2.1 cP) had a core loading of 25 ± 1.8 wt %. Meanwhile, [EMIM][BF_4_]/EG (viscosity of 10.3 ± 0.7 cP) had the highest core loading of 63 ± 0.9 wt %.

### Volatility Measurements

**Figure 4:**
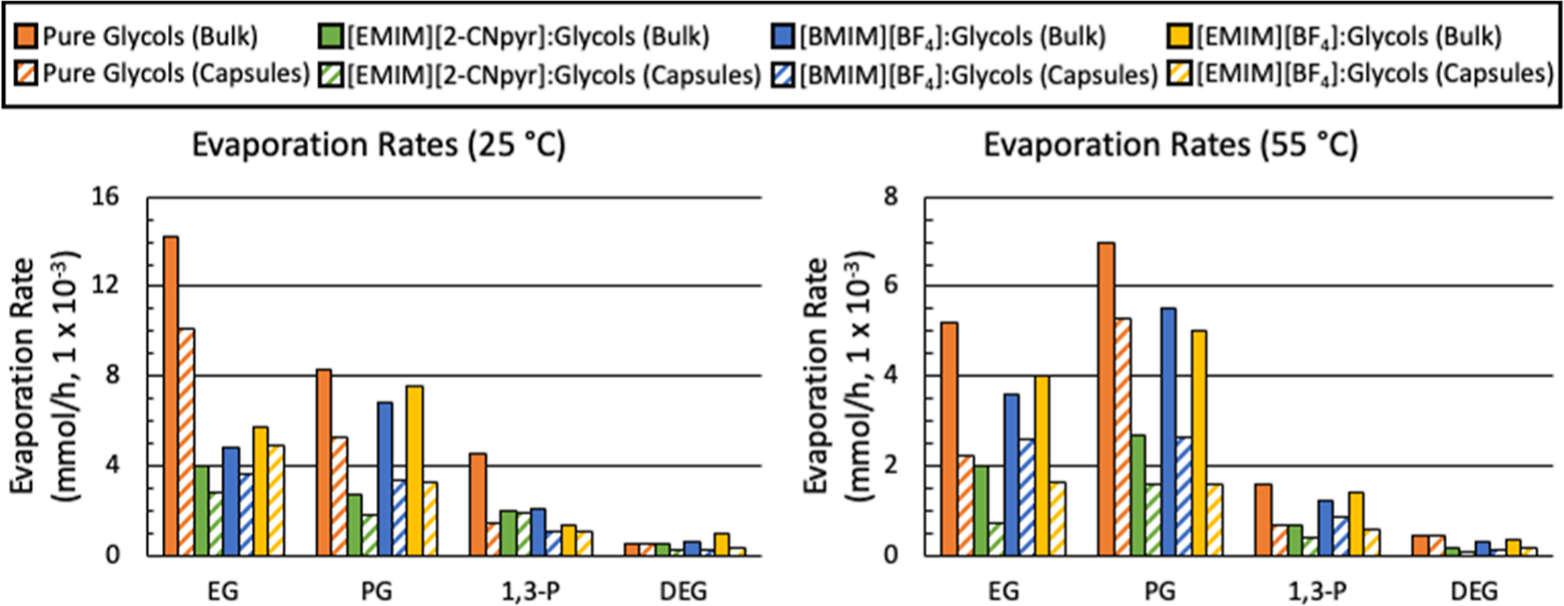
Evaporation rates of bulk liquids (solid) and encapsulated liquids (striped) at (A) 25 °C and (B) 55 °C all under a pure N_2_ environment using TGA. The slope of the last 10 min of the isotherms was determined and converted to mmol/h. To enable a comparison between a bulk and encapsulated sample, the molar quantity was held constant for each of the experiments.

Volatility of the bulk and encapsulated liquids was examined by determining the evaporation rates using thermogravimetric analysis (TGA). First, isothermal measurements under N_2_-feed were established for all systems at proposed DAC operating conditions of 25 and 55 °C, using a fresh sample for each. As previously documented, the pure ILs have no detectable evaporation rate at either temperature after minimal initial weight loss by desorption of moisture or previously absorbed gases (Figure S5).^14,62^ The evaporation rates for the glycols and IL/glycol mixtures were established by taking the slope of the last 10 min of the 1 h isothermal measurement with the assumption that evaporation was due to loss of the glycol component. The evaporation rate expressed in mmol/h can be found in Table 1 and graphically in Figure 4.

**Table 1:**
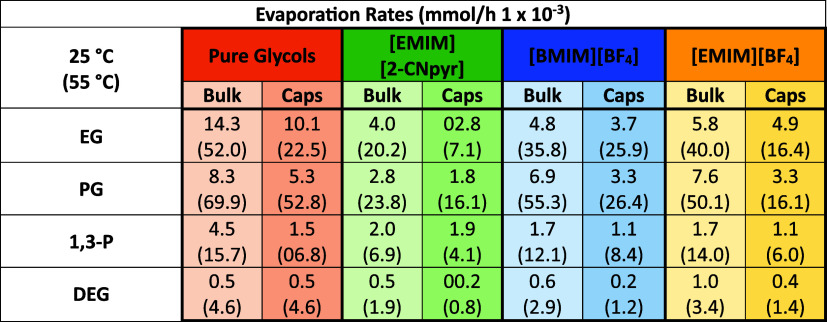
Evaporation Rates of Bulk and Encapsulated Pure Glycols and Their Mixtures at 25 and 55 °C, with the Values at 55 °C in Parentheses

The dependence of evaporation rates of the bulk glycols and their mixtures with ILs had a general trend of PG > EG > 1,3-P > DEG, which correlates with reported vapor pressures of the pure glycols.^63−65^ The only exceptions were EG and PG at 25 °C, which could be the result of the higher water content of the EG-containing samples. According to Raoult’s Law for the volatility of ideal binary mixtures (i.e., no intermolecular interactions), the vapor pressure of a component can be calculated as in eq 4
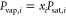
4where *x*_*i*_ is the mole fraction of a given component *i* and *P*_sat,*i*_ is saturation pressure of that component when pure. While exact vapor pressures of the mixtures cannot be estimated in TGA due to the dynamic nature of the measurements, we can estimate that evaporation rate should roughly scale with vapor pressure given that the same sample mass, instrument, pan, and conditions were used.^66^ Thus, for a mole ratio of 1:2 IL/glycol (*x*_HBD_ = 0.67), the evaporation rate of the glycol from each mixture should decrease by about 33% compared with the pure glycol, assuming a uniform sample composition. Using the results at 55 °C (faster mass loss was more reliably measurable), the volatility of glycol in the mixtures with all three ILs decreased, with 56–66% for [EMIM][2Cnpyr], 21–37% for [BMIM][BF_4_], and 11–28% for [EMIM][BF_4_]. These results support the proposed trend of relative intermolecular interaction strength: [EMIM][2-CNpyr] forming strong intermolecular interactions between the polar components (e.g., ion–dipole and dipole–dipole interactions) whereas [EMIM][BF_4_] and [BMIM][BF_4_] have weakened intermolecular interactions in the mixtures.^67^ Notably, the assumption of a uniform sample composition is likely inaccurate as diffusion limitations of the glycol in the IL may contribute to lower evaporation rates in mixtures compared to the pure glycol. Diffusion of glycols to the surface is slowed by the hydrogen bonding network, which will affect concentration of the glycol at the surface, which determines the rate of mass transfer. Therefore, the evaporation rates for [EMIM][2-CNpyr] mixtures being significantly below “ideal” could be due to a combination of higher viscosity and stronger intermolecular interactions. Notably, both DEG and IL/DEG mixtures had almost negligible evaporation rates at 25 °C (0.5–1.0 × 10^–3^ mmol/h).

The encapsulated glycols and their IL mixtures presented a further reduction in evaporation rate compared to the bulk analogs. This is particularly remarkable as the gas-contacting surface area to volume ratio is dramatically increased in the encapsulated material (∼40 cm^2^/mL for a 20 μL liquid disk with 1 cm diameter vs 850–6000 cm^2^/mL for spheres of 70–10 μm diameter). At 25 °C the general evaporation rate trend was EG > PG > 1,3-P > DEG. Contrary to the bulk analogue, the encapsulated IL mixture evaporation rate trend was different. For the IL/EG mixtures, the evaporation rates were [EMIM][BF_4_] > [BMIM][BF_4_] > [EMIM][2-CNpyr]. However, IL/PG and IL/1,3-P had evaporation rates of [EMIM][BF_4_] = [BMIM][BF_4_] > [EMIM][2-CNpyr] and [EMIM][2-CNpyr] > [EMIM][BF_4_] = [BMIM][BF_4_], respectively. Lastly, IL/DEG had an evaporation rate trend of [EMIM][BF_4_] > [BMIM][BF_4_] = [EMIM][2-CNpyr]. To be clear, [EMIM][2-CNpyr]/glycol mixtures tended to have the lowest evaporation rates, however, [EMIM][2-CNpyr]/1,3-P had a higher evaporation rate compared to pure 1,3-P and the other IL/1,3-P mixtures. This is likely due to poor capsule formation which led to core leakage during the handling of the capsules. Examining the 55 °C isothermal measurement for the capsules, the pure encapsulated glycols had a general evaporation rate trend of PG > EG > 1,3-P > DEG. Interestingly, the encapsulated IL mixture evaporation rate trend was different at 55 °C compared to 25 °C where [BMIM][BF_4_] > [EMIM][BF_4_] > [EMIM][2-CNpyr]. The only exception was with IL/DEG with the trend being [EMIM][BF_4_] > [BMIM][BF_4_] > [EMIM][2-CNpyr].

Comparing the overall bulk and encapsulated evaporation rates of the pure glycols to those of the IL/glycol mixtures, the mixtures exhibited a decrease in evaporation by an average of 36 and 40% at 25 and 55 °C, respectively. [EMIM][2-CNpyr]/glycol mixture evaporation rates decreased by 45 and 62% compared with the pure glycol evaporation rates at 25 and 55 °C, respectively. The mixtures containing DEG had the largest decrease in evaporation with encapsulation by 47 and 72% at 25 and 55 °C, respectively. These results suggest that introducing a glycol as a viscosity modifier can reduce the viscosity of the active liquid while encapsulation lowers the volatility of the core liquid, the latter of which can increase the working life. Building upon these results, capsules of [EMIM][2-CNpyr]/1,3-P and [EMIM][2-CNpyr]/DEG were selected for CO_2_ sorption and thermal desorption studies because they had the lowest evaporation rates of all systems evaluated.

### CO_2_ Uptake Experiments

**Figure 5:**
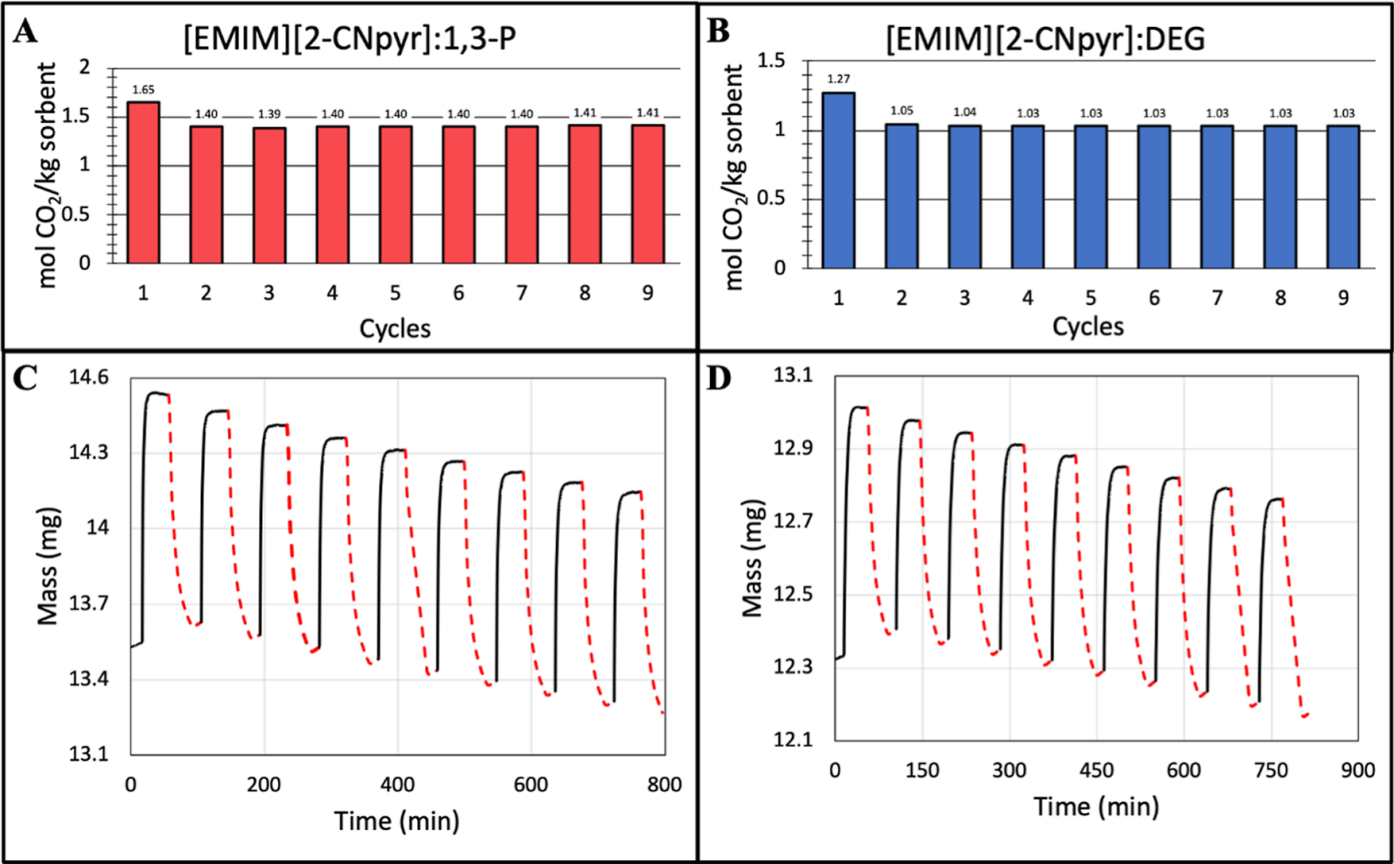
CO_2_ sorption and desorption experiments were conducted using TGA. [EMIM][2-CNpyr]/1,3-P results showed (A) the CO_2_ capacity per cycle (red bar graph) and (C) the sorption/desorption curves based on mass where the absorption (solid black) is under a pure CO_2_ environment at 25 °C and desorption (dashed red) is under a N_2_ environment at 55 °C. [EMIM][2-CNpyr]DEG results are outlined by (B) the CO_2_ capacity per cycle (blue bar graph) and (D) the sorption/desorption curves based on mass with the same conditions as (C).

Unencapsulated mixtures of [EMIM][2-CNpyr]/1,3-P and [EMIM][2-CNpyr]/DEG were saturated with CO_2_ using TGA at 25 °C under pure CO_2_ at 1 bar (Figure S6), where the CO_2_ capacities were determined to be 2.02 and 1.83 mol of CO_2_/kg of sorbent, respectively. To confirm the CO_2_ binding mechanism, ^1^H NMR spectra of the bulk liquids were taken before and after CO_2_ absorption in TGA at 25 °C under pure CO_2_ at 1 bar (Figures S7 and S8). A significant amount of CO_2_ is bound to the glycol component in both mixtures: ∼0.45 mol/mol IL in the case of DEG, and ∼0.7 mol/mol IL in the case of 1,3-P. Further, some CO_2_ binding to the cation of the IL is observed (∼0.1 and 0.05 mol/mol IL for DEG and 1,3-P, respectively). CO_2_ to the anion and water cannot be observed by proton NMR, but should constitute the remainder of the absorbed CO_2_, as water can be seen in both mixtures after absorption with a broad peak around 5–6 ppm. Assumptions for these calculations are described in the respective figure captions in the Supporting Information. CO_2_ sorption for the encapsulated IL/glycols was performed at 25 °C with a pure CO_2_-gas stream and thermal desorption was performed at 55 °C under a N_2_ gas stream over nine sorption–desorption cycles. The first cycle was used to condition the material and determine the sorption and desorption times. Figure 5 shows the sorption–desorption cycles for [EMIM][2-CNpyr]/1,3-P and [EMIM][2-CNpyr]/DEG capsules. For both systems, a slight decrease in capacity is observed after the first cycle, which may be attributed to the formation of bicarbonate with trace water in the mixture. However, no significant CO_2_ capacity is lost during subsequent cycles with capacities at 1.40 and 1.03 mol of CO_2_/kg of sorbent for [EMIM][2-CNpyr]/1,3-P and [EMIM][2-CNpyr]/DEG, respectively (Figure 5A,B). The difference in CO_2_ capacity from the saturated mixtures and the capsules can be attributed primarily due to low core loading wt %, and some evaporation of the core liquid likely occurred, which is supported by a decrease in the baseline mass with each cycle (Figure 5C,D). The average rate loss of sample mass after the initial cycle was determined to be 0.62 × 10^–3^ and 0.32 × 10^–3^ mmol/h for [EMIM][2-CNpyr]/1,3-P and [EMIM][2-CNpyr]/DEG, respectively. This was lower than expected based on the evaporation rate of the capsules under a 1 h isotherm, as discussed above (4.1 × 10^–3^ and 0.8 × 10^–3^ mmol/h at 55 °C, respectively).

### Breakthrough Experiments

To assess the sorbent performance of [EMIM][2-CNpyr], [EMIM][2-CNpyr]/DEG, and [EMIM][2-CNpyr]/1,3-P capsules and rate limitations (e.g., diffusion and reaction kinetics) in DAC, breakthrough analyses were conducted at 25 °C under 1 bar of N_2_ with 500 ppm of CO_2_. In a typical breakthrough experiment, capsules were packed in a column such that gas could be flowed through and the concentration of CO_2_ coming out of the column was measured. An ideal sorbent, i.e., one with no rate limitations, will present a step curve, where all CO_2_ in the gas stream is absorbed until the sorbent reaches saturation, at which point the CO_2_ concentration in the effluent will increase to the feed concentration. The breakthrough time (t_BT_) is determined when the effluent concentration reaches 5% of the feed concentration (i.e., 25 ppm of CO_2_). Sorbents with greater mass transport limitations will present elongated S-curves, where elongation increases with greater kinetic barriers.

**Figure 6:**
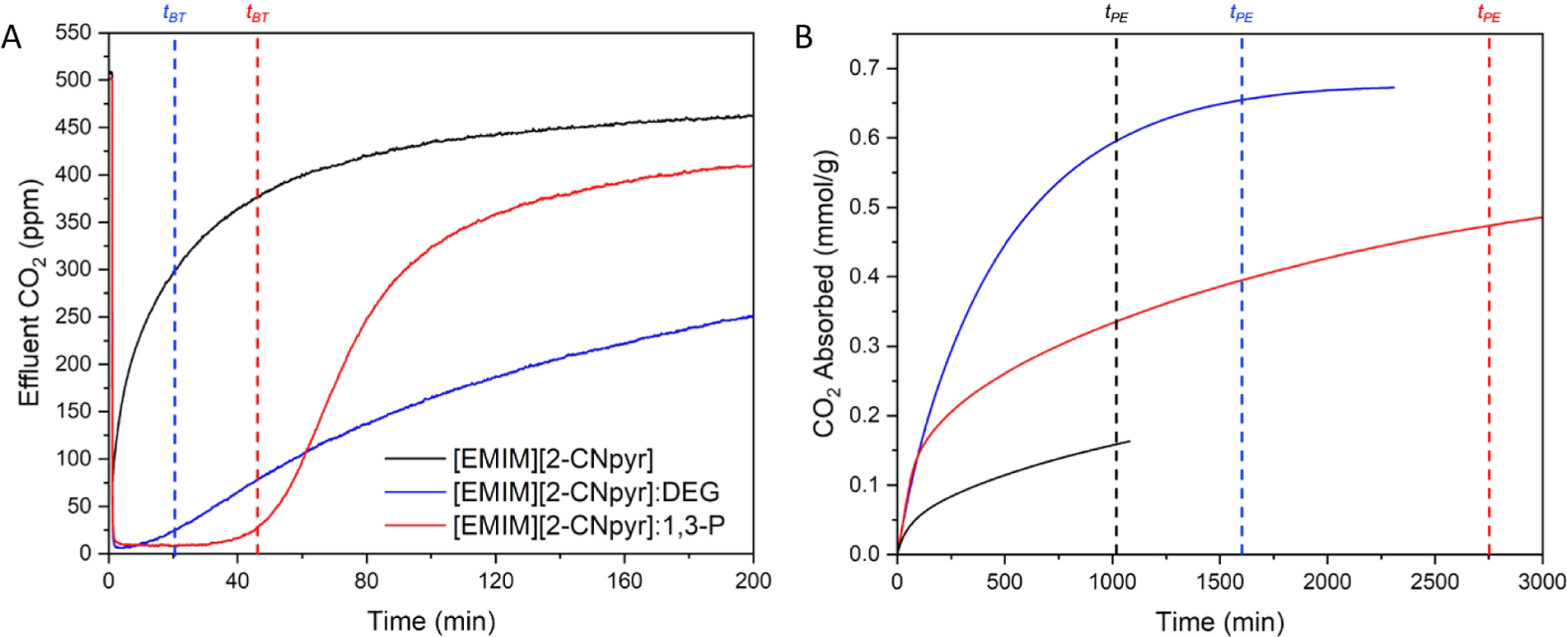
Results of breakthrough experiments for capsules with [EMIM][2-CNpyr]-based cores. (A) First 200 min of breakthrough curves, with vertical dashed lines signifying the breakthrough time for each capsule. (B) CO_2_ loading of the capsules calculated from the effluent CO_2_ concentration using eq 2. Vertical dashed lines signify the pseudoequilibrium time.

All three capsule types showed rate limitations such that by integration of the curves with respect to the feed concentration from 0 min to *t*_BT_ and *t*_PE_, the breakthrough capacity and the pseudoequilibrium capacity can be determined, respectively. The first 200 min of breakthrough curves for capsules of [EMIM][2-CNpyr], [EMIM][2-CNpyr]/DEG, and [EMIM][2-CNpyr]/1,3-P at 500 ppm of CO_2_ are presented in Figure 6A. The CO_2_ loading over time is plotted in Figure 6B. Capsules containing only [EMIM][2-CNpyr] in the core were severely rate limited and did not absorb all CO_2_ in the feed at the start of the experiment. This may be due to the low core loading (25 ± 1.8 wt %), however, this was comparable to the [EMIM][2-CNpyr]/1,3-P (28 ± 1.6 wt %) and [EMIM][2-CNpyr]/DEG (34 ± 3.6 wt %). Another possible cause for the limited breakthrough time could be column packing, as these capsules were highly aggregated compared to the [EMIM][2-CNpyr]/DEG and [EMIM][2-CNpyr]/1,3-P capsules, which made it difficult to create a uniformly packed column without breaking the capsules. Aggregation also yields larger effective particles, thereby increasing diffusion distance and leading to greater rate limitations. The higher viscosity of pure [EMIM][2-CNpyr] (75.3 ± 2.1 cP) compared to the glycol mixtures of [EMIM][2-CNpyr]/1,3-P (51.1 ± 2.1 cP) and [EMIM][2-CNpyr]/DEG (55.8 ± 2.4 cP) is also likely a significant factor impacting the sorption rate, especially in combination with capsule aggregation. The ratio of CO_2_ to [EMIM][2-CNpyr] at full capacity is consistent with isotherm sorption values for bulk [EMIM][2-CNpyr] at 50 Pa (∼500 ppm).^14^ Capsules containing a [EMIM][2-CNpyr]/1,3-P mixture had a *t*_BT_ of 44 min, indicating significantly reduced kinetic limitations compared to the capsules of pure IL. These capsules were a finer powder which made uniform packing feasible. The pseudoequilibrium capacity was significantly higher as well, which is consistent with previous reports in a mixture of [EMIM][2-CNpyr] with EG, where the ratio of CO_2_ to [EMIM][2-CNpyr] at lower partial pressures dramatically increases upon mixing with an HBD due to an additional “carbonate” binding route, where the hydroxyl is deprotonated by the basic IL anion as reported by Lee et al.^19^

Interestingly, capsules of [EMIM][2-CNpyr]/DEG had a lower breakthrough time, 19 min, as well as a lower time to pseudoequilibrium, and yet displayed a much higher pseudoequilibrium capacity compared to the [EMIM][2-CNpyr]/1,3-P capsules. This is likely due to the [EMIM][2-CNpyr]/1,3-P capsules losing some 1,3-P over the extended sorption cycle, reducing the impact of carbonate formation and increasing the diffusion resistance for CO_2_ by increasing viscosity. This is not observed to the same extent in the [EMIM][2-CNpyr]/DEG due to the higher boiling point of DEG, leading to greater overall CO_2_ capacity of the mixture at low partial pressure, despite greater diffusion limitations. Both capsules were observed to be fine powders and were easily packed into the column, and thus, the packing density was assumed to be similar.

Since all the major CO_2_ binding routes in the liquids of the capsule core involve the deactivation of a [2CNpyr] anion, we can represent “binding site saturation” of the liquid as the mole ratio of CO_2_ to IL. Capsules of pure [EMIM][2-CNpyr] had a breakthrough CO_2_ capacity of 0 due to significant kinetic barriers and packing limitations, in which the measured effluent CO_2_ concentration never reached 25 ppm. In contrast, capsules of [EMIM][2-CNpyr]/1,3-P had the largest breakthrough CO_2_ capacity (0.08 mol of CO_2_/kg of sorbent), despite having the longest pseudoequilibrium time (2756 min). [EMIM][2-CNpyr]/DEG capsules had a lower breakthrough CO_2_ capacity of 0.03 mol of CO_2_/kg of sorbent with almost half the pseudoequilibrium time (1637 min) compared to [EMIM][2-CNpyr]/1,3-P capsules. Interestingly, [EMIM][2-CNpyr]/DEG had the highest pseudoequilibrium CO_2_ capacity (0.66 mol of CO_2_/kg of sorbent), which suggests a higher binding site saturation of 0.75 compared to 0.35 and 0.13 for [EMIM][2-CNpyr]/1,3-P and [EMIM][2-CNpyr] capsules, respectively. Although the core loading wt % is lower with capsules of pure [EMIM][2-CNpyr] compared to the capsules of the IL/glycol mixtures, pure [EMIM][2-CNpyr] capsules and [EMIM][2-CNpyr]/1,3-P capsules had similar core loading wt % (25 ± 1.8 and 28 ± 1.6 wt %, respectively), yet the pseudoequilibrium CO_2_ capacity was much higher for [EMIM][2-CNpyr]/1,3-P. Table 2 shows the pseudoequilibrium values from breakthrough experiments compared with other IL-based materials that have been reported near DAC conditions. Some promising candidates in other material classes such as metal organic frameworks (MOFs) and zeolites are also included for a comprehensive comparison.

**Table 2:** Capacity Results in the Pressure Range Relevant to DAC (<5000 ppm) for the Materials Presented in This Study^a^^,^^b^

material	core loading	*P*_CO2_ (mbar)	temp (°C)	balance gas	regen temp (°C)	RH (%)	CO_2_ capacity (mol CO_2_/kg sorbent)	ref
[EMIM][2-CNpyr]@PU	25 wt %	0.5	25	N_2_	55	0	0.16	This study
[EMIM][2-CNpyr]/1,3-P@PU	28 wt %						0.47	
[EMIM][2-CNpyr]/DEG@PU	34 wt %						0.66	
[EMIM][2-CNpyr]@PU	60 wt %	5	25	N_2_		0	0.82	(14)
						100	0.9	
[EMIM][2-CNpyr]		0.4	25	N_2_, air^c^	60	0	0.75	(13)
		2.5					1.8	
		5					2.3	(19)
		0.4			80	40	1.75	(68)
		2.5					2.9	
		5					3.6	
[EMIM][2-CNpyr]/EG (1:2)		0.4	25	N_2_	40	0	0.95	(19)
		2					2.08	
		5					2.35	
[AEEA][Tf_2_N]/[EMIM][AcO]		1	50	none		0	0.157	(69)
[Ch][Pro]/EG (1:2)		5	25	N_2_			1	(52)
[Ch][Trz]/EG (1:2)		5	25	N_2_	50		0.4	(70)
NPEI@SIP	50 wt %	0.4	25	N_2_	120	0	1.05	(71)
			25	N_2_		80	1.66	
SIL/Ni-MOF	0.48:1 SIL/Ni^d^	0.4	25	He	80	2.5	0.58	(72)
En-Mg_2_ (dobpdc)		0.39	25	air	150	0	2.68	(73)
biomass PEI hydrogel	25 wt %	0.4	25	N_2_	60	70^e^	3.6	(74)
PEI/MIL-101(Cr)	60 wt %	0.4	25	He	110	0	1.35	(75)
Li-LSX powder		0.4	25	air	240	0	0.82	(76)
Zeolite 13X		5	25	N_2_	350	0	0.79	(14)

a Other IL-based materials, as well as non-IL materials, from the literature are included for comparison.

b Core@shell material, PU—polyurea, AEEA—*N*-(2-aminoethyl)ethanolamine, AcO—acetate, Ch—choline, Pro—prolinate, Trz—1,2,4-triazolate, PEI—polyethylenimine, NPEI—nanoparticle organic hybrid material w/PEI (PEI on silica), SIP—solvent impregnated polymer, SIL—superbase-derived IL, En-Mg2(dobpdc)—ethylene diamine functionalized MOF, Li-LSX—lithium-substituted low-silica zeolite.

c No measurable difference in capacity was observed between air and N_2_ balance.

d Mol of SIL per mol Ni.

e Sample pretreated at 70% RH.

## Conclusions

The addition of glycols to ILs resulted in decreased viscosity enabling improve CO_2_ transport rates while the encapsulation of these mixtures mitigated the evaporation of the glycols under conditions relevant for DAC of CO_2_. The decrease in the evaporation rate is mainly attributed to the diffusion limitations of the glycols through the IL and molecular interactions (e.g., hydrogen bonding, etc.). Encapsulation via a soft-templated approach demonstrated a substantial method to reduce glycol evaporation by about 40%, while also maintaining CO_2_ permeability and increased surface area for CO_2_ sorption. The capsules of the two mixtures which had the lowest evaporation rate were used for CO_2_ sorption, specifically capsules of the task specific IL, [EMIM][2-CNpyr], and DEG or 1,3-P. Capsules of [EMIM][2-CNpyr]/DEG had the highest CO_2_ capacity but a large kinetic barrier, as determined by sorption/desorption cycles and breakthrough experiments. This work expands the approaches of using ILs for carbon capture; viscosity modifiers used enhance the rate of CO_2_ uptake can be volatile, but with encapsulation this volatility is mitigated. This approach may greatly extend the life span of DAC materials. Ongoing work in our laboratories addresses further tuning the composition of the capsules (both core and shell), as well as composite structure, for enhanced performance in DAC of CO_2_ in addition to other separations and energy management.

## Supporting Information Available

Additional characterization of capsule composition and thermal stability (PDF)

## Abbreviations

DAC: direct air capture
IL: ionic liquid
TSIL: task specific ionic liquid
EG: ethylene glycol
1, 3-P: 1,3-propanediol
PG: propylene glycol
DEG: diethylene glycol
[EMIM][2-CNpyr]: 1-ethyl-3-methylimidazolium 2-cyanopyrrolide
[BMIM][BF4]: 1-butyl-3-methylimidazolium tetrafluoroborate
[EMIM][BF4]: 1-ethyl-3-methylimidazolium tetrafluoroborate
DAPM: 4, 4′-diaminodiphenylmethane
HDI: hexamethylene diisocyanate
GO: graphene oxide
TGA: thermogravimetric analysis
