# Supplementary material for: Ionic Liquid–Glycol Mixtures for Direct Air Capture of CO2: Decreased Viscosity and Mitigation of Evaporation Via Encapsulation
Source: ACS Sustain Chem Eng. 2024 May 7;12(20):7882–93. doi: 10.1021/acssuschemeng.4c01265 (PMC11110104; doi:10.1021/acssuschemeng.4c01265)
Supplement: Supplementary file 1 — sc4c01265_si_001.pdf [file sc4c01265_si_001.pdf]

Supporting Information for:

Ionic liquid-glycol mixtures for direct air capture of CO_2: decreased viscosity and mitigation of evaporation via encapsulation

Cameron D.L. Taylor,[†,1] Aidan Klemm, [†,2] Luma Al-Mahbobi,[1] B. Jack Bradford,[1] Burcu Gurkan,[2,*] Emily B. Pentzer[1,3,*]

[1]*Department of Materials Science and Engineering, Texas A&M University, College Station, Texas 77843, USA*
[2]*Department of Chemical Engineering Biomolecular Engineering, Case Western Reserve University, Cleveland, Ohio 44106, USA*
[3]*Department of Chemistry, Texas A&M University, College Station, Texas 77843, USA*
† These authors contributed equally
*Corresponding Authors: beg23@case.edu; emilypentzer@tamu.edu

Figures & Tables

Fig. S1. ^{1}H- NMR spectrum example for core wt% analysis of [BMIM][BF_4]:DEG with a standard of mesitylene in DMSO-d6. 3

Fig. S2. Example of thermal decomposition of capsules and bulk materials using TGA. 3

Fig. S3. Optical microscopy images of the emulsions with a continuous phase of octane/mineral oil (1:1, v/v). 5

Fig. S4. SEM images of isolated capsules captured at a voltage of 10-20 kV with a sputter-coating of 10 nm of Au. 6

Fig. S5. Thermal decomposition of pure ILs using TGA. 6

Fig. S6. CO_2 saturation using TGA of unencapsulated mixtures of [EMIM][2-CNpyr]:1,3-P (top) and [EMIM][2-CNpyr]:DEG (bottom). Excess water or gasses absorbed were removed from the capsule using an isothermal step at 55 °C for 30 min followed by an isothermal step at 25 °C for 25 min under an N_2 atmosphere (25 mL/min). The absorption was conducted at 25 °C in pure CO_2 until the slope of the mass curve did not change. 7

Fig. S7. ^{1}H-NMR spectra of [EMIM][2-CNpyr]:DEG in $CDCl_3$ before (top) and after (bottom) CO_2 absorption under pure CO_2 at 1 bar, 25 ˚C. Interestingly, [EMIM] peaks downshift by ~0.17 ppm after CO_2 after absorption, likely due to change in the pH of the solution. [EMIM]-CO_2 amount determined via integration of peak c' divided by 2, ~0.1 mol / mol IL. DEG-CO_2 binding ratio determined by integration of peak 2'-3 divided by 4, ~0.45 mol / mol IL. Results correlate with other overlapping peak integrations. 8

Fig. S8. ^{1}H-NMR spectra of [EMIM][2-CNpyr]:1,3-P in $CDCl_3$ before (top) and after (bottom) CO_2 absorption under pure CO_2 at 1 bar, 25 ˚C. Interestingly, [EMIM] peaks downshift by ~0.17 ppm after CO_2 after absorption, likely due to change in the pH of the solution. [EMIM]-CO_2 amount determined via integration of peak c' divided by 2, ~0.05 mol / mol IL. DEG-CO_2 binding ratio determined by integration of peak 2' divided by 2, ~0.7 mol / mol IL. Results correlate with other overlapping peak integrations. 9

Table S1. Measured viscosity and density of mixtures with ionic liquids and EG (ethylene glycol), PG (propylene glycol), 1,3-PD (1,3-propanediol), and DG (diethylene glycol). 4

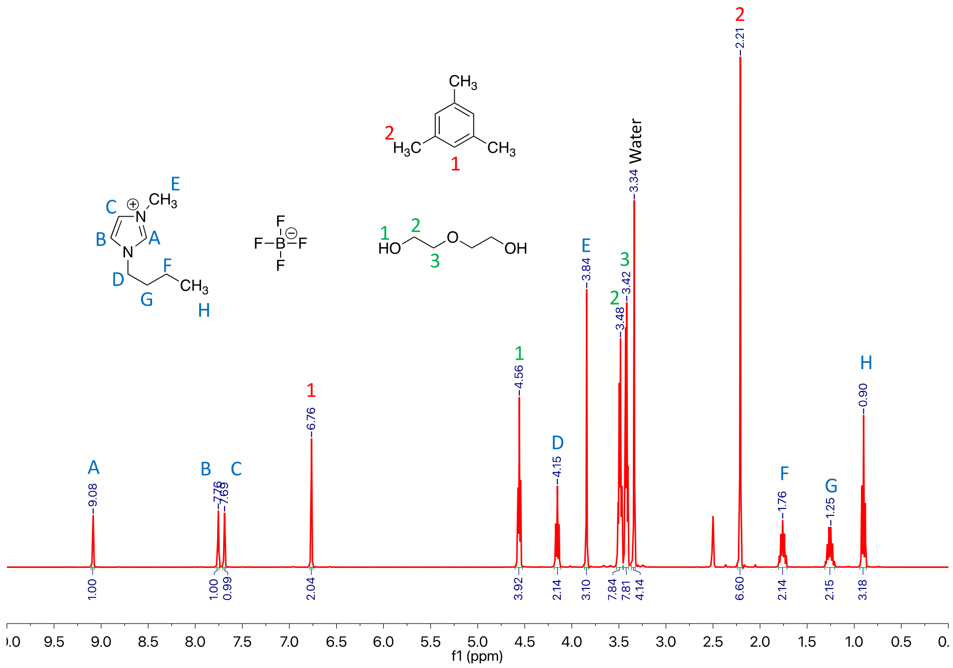

Fig. S1. ^{1}H NMR spectrum example for core wt% analysis of [BMIM][BF_4]:DEG with a standard of mesitylene in DMSO-d6.

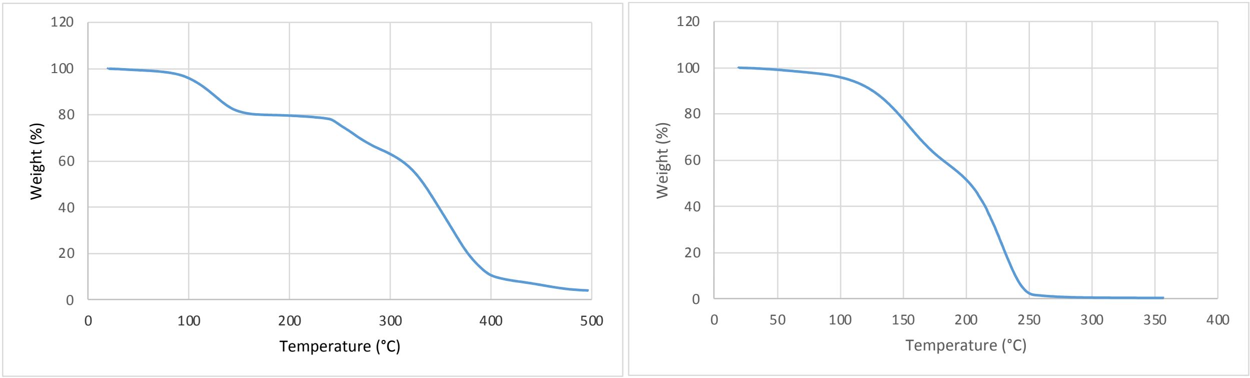

BMIMBF4:1,3P
Caps

BMIMBF4:1,3P
Bulk

Fig. S2. Example of thermal decomposition of capsules and bulk materials using TGA.

Table S1. Measured viscosity and density of mixtures with ionic liquids and EG (ethylene glycol), PG (propylene glycol), 1,3-PD (1,3-propanediol), and DG (diethylene glycol).

Mixture		Molar Ratio	Viscosity (cP)	Density (g/cm^3 ±0.00001)	Water Content (PPM)	Excess Molar Volume	Capsule Diameter (µm)	Core wt%
Glycol	IL							
No Glycols	[EMIM] [2-CNpyr]	-	75.3 ± 2.1	1.08681	-	-	41 ± 18	25 ± 1.8
	$[EMIM][BF_4]$	-	21.6 ± 6.2	1.28200 (1.26522 at 40 °C)	-	-	35 ± 13	69 ± 1.6
	$[BMIM][BF_4]$	-	73.0 ± 8.5	1.20300 (1.1899 at 40 °C)	-	-	45 ± 18	73 ± 1.1
EG	Pure EG	-	14.7 ± 0.2	1.10976	-	-	33 ± 11	60 ± 3.2
	[EMIM] [2-CNpyr]	1:2	34.2 ± 2.8	1.10346	7350.2	-0.72315	47 ± 12	35 ± 2.2
	$[EMIM][BF_4]$	1:2	8.7 ± 0.4	1.20296	7290.7	0.69434	17 ± 6	63 ± 0.9
	$[BMIM][BF_4]$	1:2	10.3 ± 0.7	1.16272	7629.3	0.47129	30 ± 11	62 ± 0.7
PG	Pure PG	-	36.6 ± 2.6	1.04000		-	55 ± 21	54 ± 0.3
	[EMIM] [2-CNpyr]	1:2	50.3 ± 3.1	1.06987	2114.9	-0.61713	52 ± 14	35 ± 0.6
	$[EMIM][BF_4]$	1:2	17.0 ± 1.0	1.16127	2118.2	0.55850	17 ± 6	57 ± 0.3
	$[BMIM][BF_4]$	1:2	22.7 ± 2.5	1.12732	2241.4	0.42642	46 ± 23	52 ± 1.8
1,3-P	Pure 1,3-P	-	35.9 ± 2.0	1.05300 (1.10370 at 40 °C)	-	-	33 ± 12	53 ± 0.3
	[EMIM] [2-CNpyr]	1:2	51.1 ± 1.5	1.07987	3860.2	-0.83220	48 ± 14	28 ± 1.6
	$[EMIM][BF_4]$	1:2	10.1 ± 0.7	1.15690*	2870.7	3.8875	20 ± 6	57 ± 0.9
	$[BMIM][BF_4]$	1:2	17.0 ± 0.4	1.12378*	3509.3	2.0571	35 ± 12	54 ± 0.8
DEG	Pure DG	-	25.9 ± 2.4	1.11800	-	-	70 ± 29	50 ± 1.2
	[EMIM] [2-CNpyr]	1:2	55.8 ± 2.4	1.10687	2486.7	-0.281	70 ± 16	34 ± 3.6
	$[EMIM][BF_4]$	1:2	11.9 ± 0.8	1.19119	1876.7	0.036047	20 ± 7	62 ± 0.9
	$[BMIM][BF_4]$	1:2	25.0 ± 1.3	1.15824	2704.9	0.86919	55 ± 28	57 ± 0.9

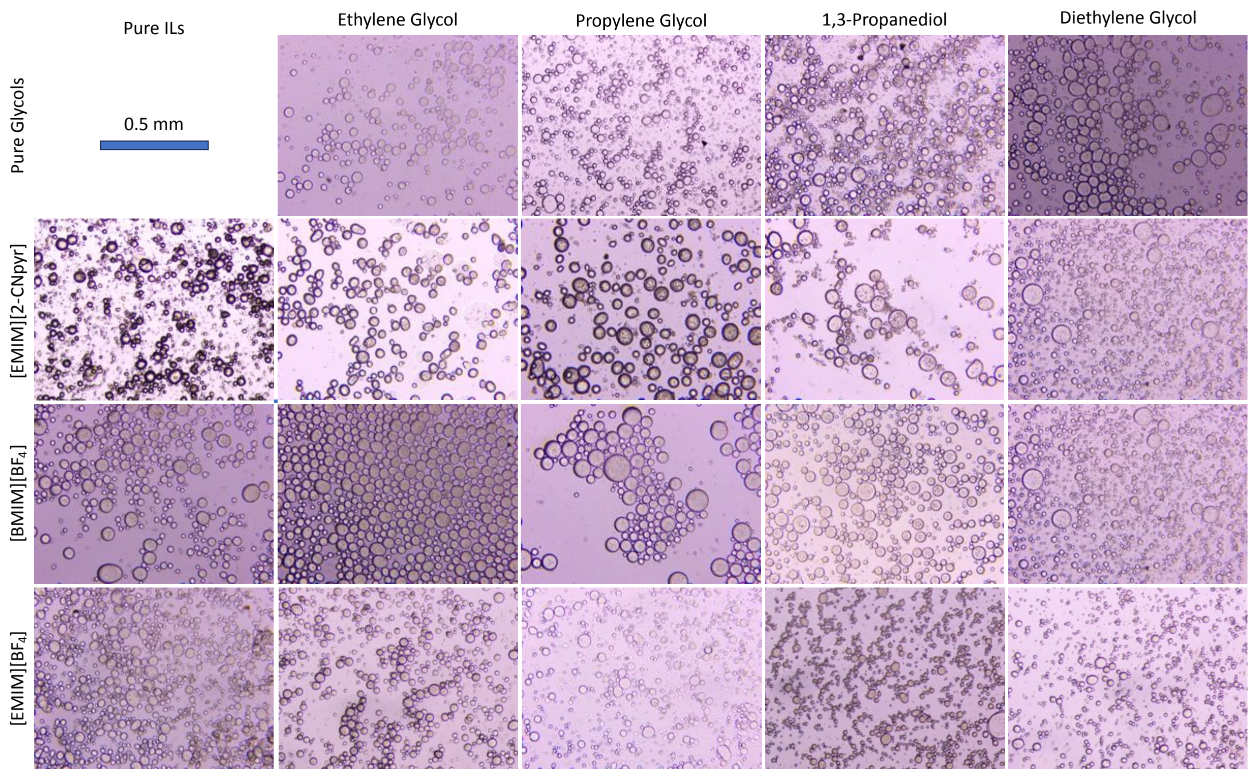

Fig. S3. Optical microscopy images of the emulsions with a continuous phase of octane/mineral oil (1:1, v/v).

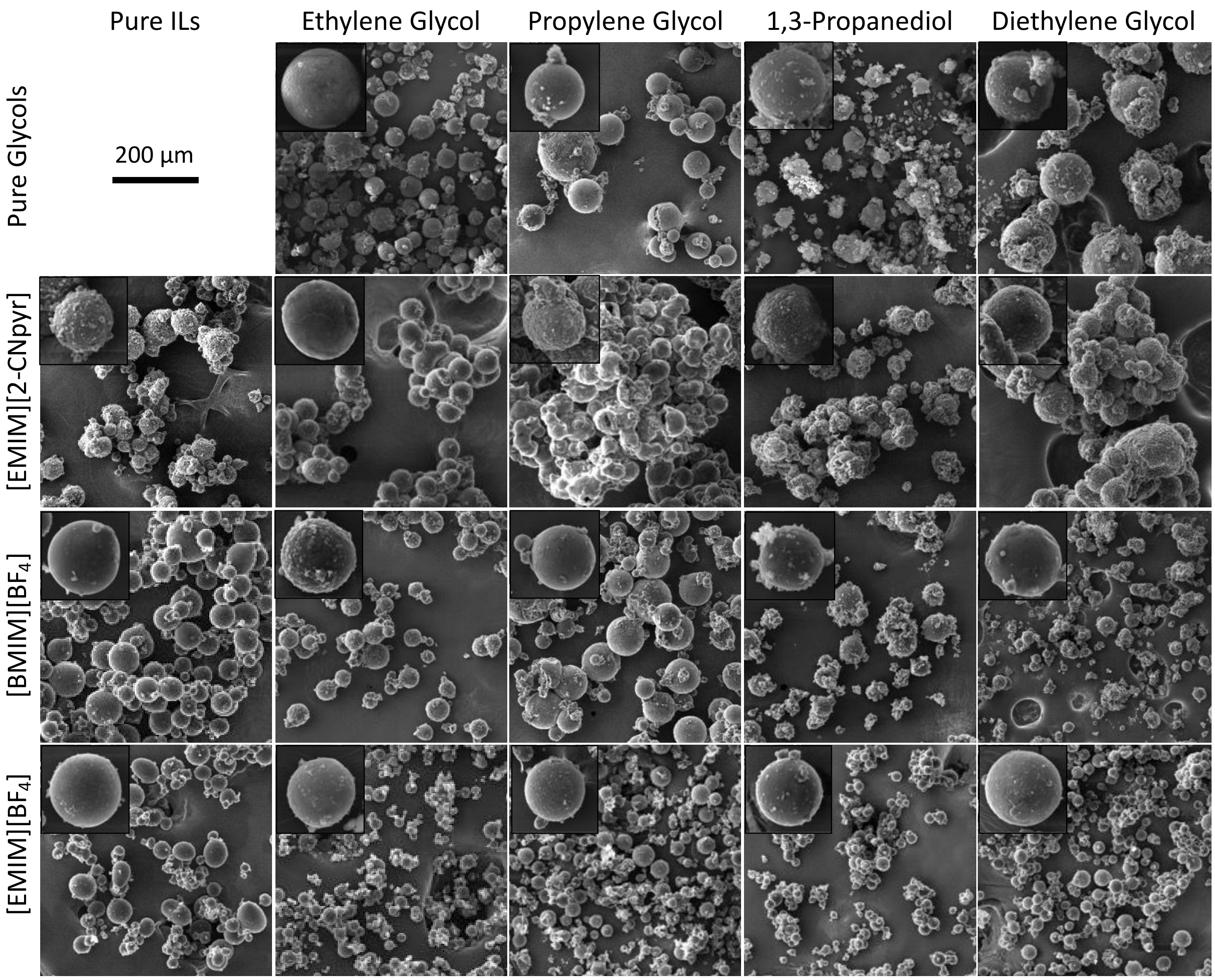

Fig. S4. SEM images of isolated capsules captured at a voltage of 10-20 kV with a sputter-coating of 10 nm of Au.

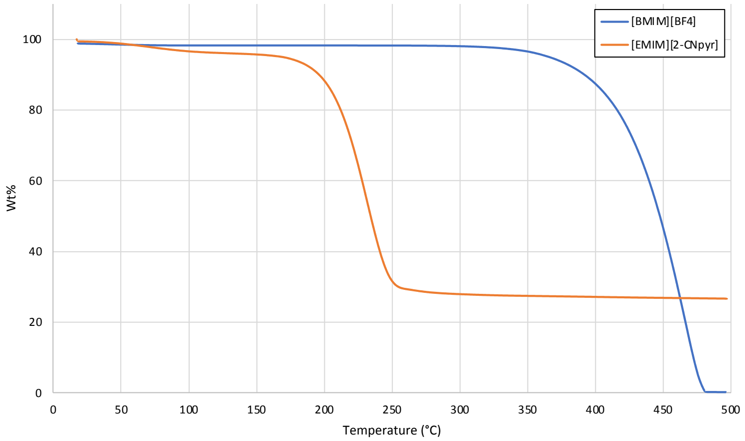

Fig. S5. Thermal decomposition of pure ILs using TGA.

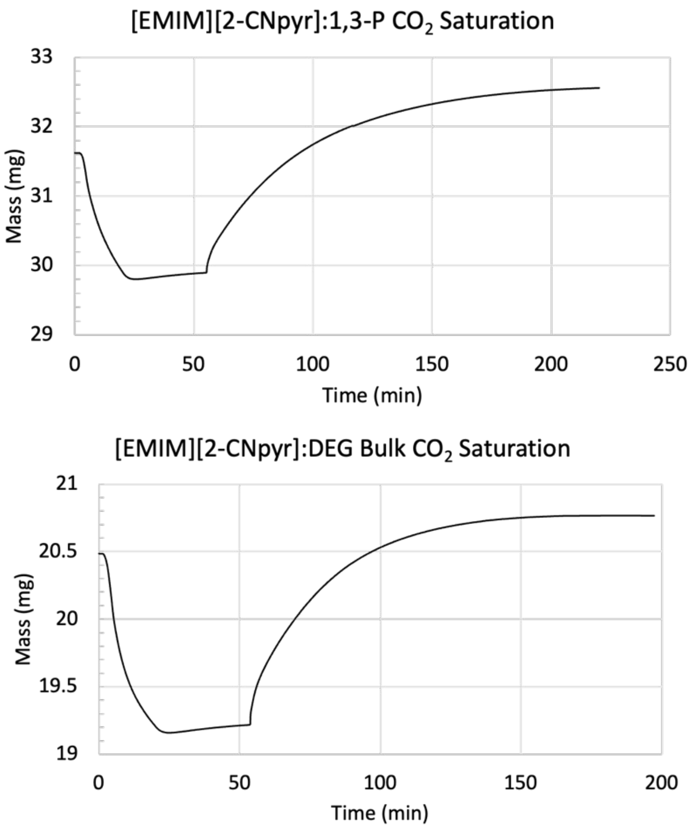

Figure S6. CO_2 saturation using TGA of unencapsulated mixtures of [EMIM][2-CNpyr]:1,3-P (top) and [EMIM][2-CNpyr]:DEG (bottom). Excess water or gasses absorbed were removed from the capsule using an isothermal step at 55 °C for 30 min followed by an isothermal step at 25 °C for 25 min under an N_2 atmosphere (25 mL/min). The absorption was conducted at 25 °C in pure CO_2 until the slope of the mass curve did not change.

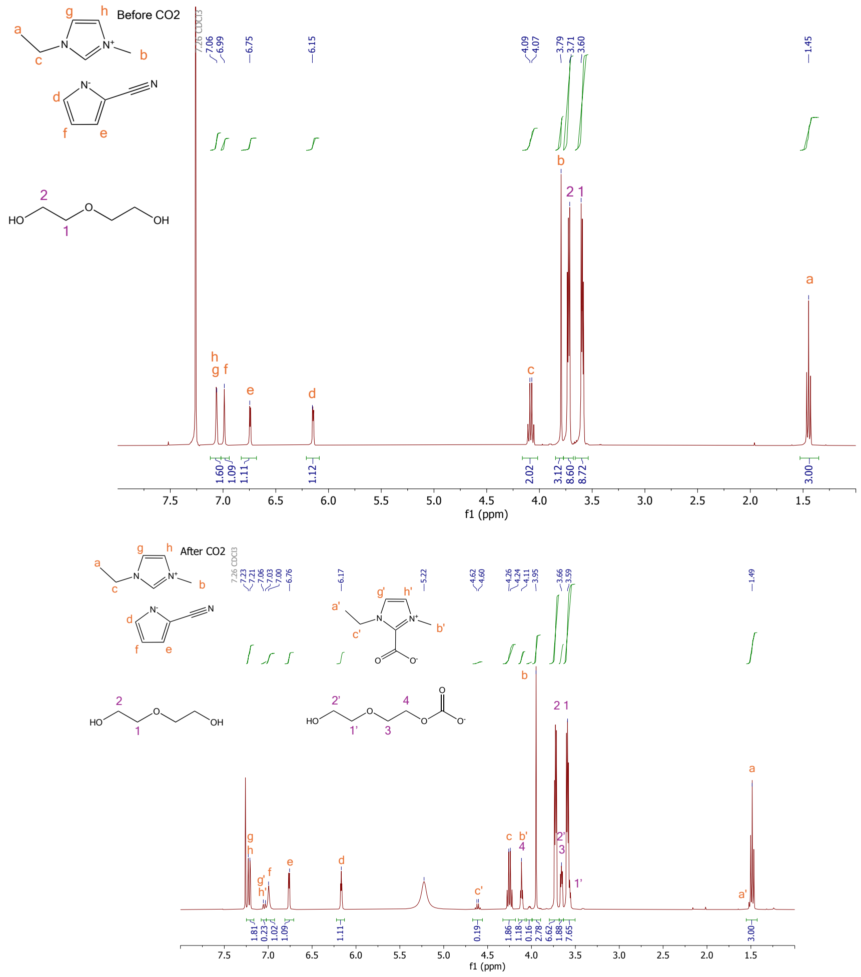

Figure S7. ^{1}H NMR spectra of [EMIM][2-CNpyr]:DEG in $CDCl_3$ before (top) and after (bottom) CO_2 absorption under pure CO_2 at 1 bar, 25 ˚C. Interestingly, [EMIM] peaks downshift by ~0.17 ppm after CO_2 after absorption, likely due to change in the pH of the solution. [EMIM]-CO_2 amount determined via integration of peak **c’** divided by 2, ~0.1 mol / mol IL. DEG-CO_2 binding ratio determined by integration of peak **2’-3** divided by 4, ~0.45 mol / mol IL. Results correlate with other overlapping peak integrations.

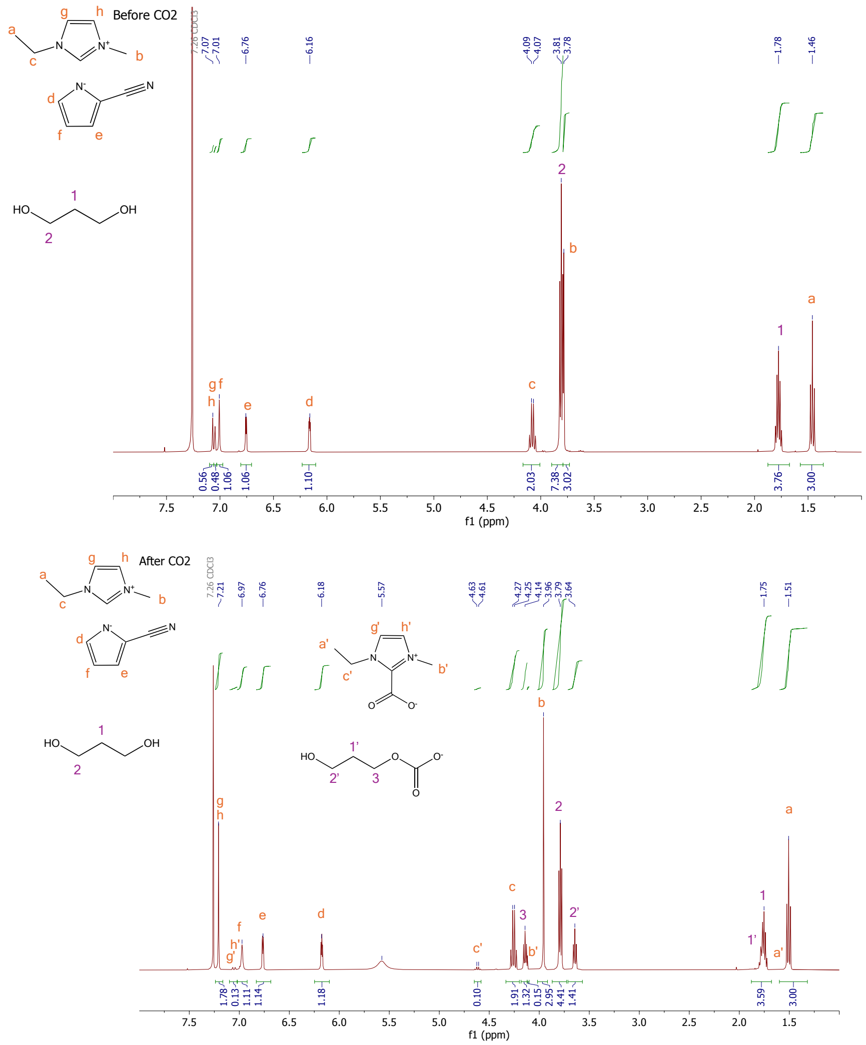

Figure S8. ^{1}H-NMR spectra of [EMIM][2-CNpyr]:1,3-P in $CDCl_3$ before (top) and after (bottom) CO_2 absorption under pure CO_2 at 1 bar, 25 ˚C. Interestingly, [EMIM] peaks downshift by ~0.17 ppm after CO_2 after absorption, likely due to change in the pH of the solution. [EMIM]-CO_2 amount determined via integration of peak **c'** divided by 2, ~0.05 mol / mol IL. DEG-CO_2 binding ratio determined by integration of peak **2'** divided by 2, ~0.7 mol / mol IL. Results correlate with other overlapping peak integrations.
